# Body trust in Korean population: validation of the Korean version of the body trust scale

**DOI:** 10.3389/fpsyt.2025.1631918

**Published:** 2025-08-21

**Authors:** Yunyoung Oh, Jang-Won Seo

**Affiliations:** Department of Psychology, Jeonbuk National University, Jeonju, Republic of Korea

**Keywords:** body trust, interoception, BTS, validity, reliability

## Abstract

**Background:**

Interoception is the ability to perceive and integrate internal sensations. A key component of interoception, body trust, involves trusting internal sensations in daily life and feeling safe within one’s own body. Problems in body trust are linked to psychological disorders, including eating disorders and suicidal behaviors. However, existing tools for measuring body trust have several limitations, and the most recently developed promising instrument to address these issues is the Body Trust Scale (BTS). This study aimed to validate the Korean version of the BTS in a sample of Korean adults.

**Methods:**

A total of 775 participants completed the BTS and other measures of positive body image, interoceptive sensibility, body investment, eating pathology, visceral sensitivity, self-harm behaviors, and suicidal ideation. The reliability, factor structure, invariance across age groups and gender, convergent validity, and discriminant validity of the BTS were examined.

**Results:**

Results revealed that the Korean version of BTS has a three-factor structure consistent with the original scale. It also demonstrated good internal consistency, as well as convergent and discriminant validity. Partial scalar invariance across age groups and full measurement invariance across gender were also established.

**Conclusion:**

The Korean version of the BTS is a reliable and valid instrument for measuring body trust in Korean adults. It is expected to have clinical utility for assessing body trust, with a specific focus on eating pathology and suicide risk.

## 1 Introduction

Interoception refers to the ability to perceive, attend to, and integrate internal bodily sensations (1), which occurs through communication between the body and brain (2). It includes various internal processes like heart rate, sensations of warmth, digestive signals (e.g., satiety, hunger), and pain experienced in the body (3). Interoception can be experienced both consciously and unconsciously (4, 5) and plays a crucial role in maintaining homeostasis (6). Garfinkel et al. (7) divided interoception into three dimensions: interoceptive accuracy, interoceptive sensibility, and interoceptive awareness. Interoceptive accuracy refers to the ability to objectively identify internal sensations. Recently, it has also been called interoceptive sensitivity (4). Interoceptive sensibility refers to the subjective belief in one’s ability to detect and process internal sensations, which can be measured through self-reports. Interoceptive awareness represents a metacognitive insight into one’s interoceptive accuracy, specifically the correspondence between physiological accuracy and subjective evaluation.

People experience interoception at every moment. However, some individuals have low interoceptive sensitivity that makes it difficult to recognize their bodily states, while others are overly sensitive and experience problems due to negatively interpreting bodily sensations (5). Such interoceptive dysfunction has been associated with various psychopathologies (2, 5, 8). For example, interoceptive dysfunction can lead to a disconnection from the body and a perception of it as an object rather than a human being, increasing the risk of self-harm behaviors (9, 10). Similarly, impairment in gastric interoception can distort the perception of satiety, potentially leading to binge eating or avoidance of food intake (11). Martin et al. (12) conducted a systematic review and revealed that interoceptive deficit is a commonly observed clinical feature in eating disorders. Thus, interoceptive dysfunction is considered a key mechanism underlying eating disorders and suicidality (13, 14).

Mehling et al. (4, 15) identified that interoceptive sensibility comprises eight dimensions, thereby demonstrating that interoception is a multidimensional construct. One of these dimensions, body trust, is particularly prominent in psychopathology (13, 16). Body trust refers to trusting one’s bodily sensations, feeling safe and comfortable in one’s body (4, 17), and valuing body ownership, which helps distinguish one’s own body from others and the external environment (16, 18).

Individuals with decreased body trust tend to rely on external cues over internal bodily signals for self-regulation (19), which may contribute to psychological problems. According to several empirical studies, diminished body trust has been associated with suicidal ideation and suicide attempts across various populations (10, 14) and has been shown to predict self-harming behaviors (9). Grunewald et al. (16) further confirmed the predictive validity of body trust in relation to suicidal ideation. Moreover, the Trusting subscale of the Multidimensional Assessment of Interoceptive Awareness (MAIA) (15) was identified as one of the factors negatively correlated with eating disorders (20, 21). Diminished body trust in eating disorder samples bridges the relationship between interoceptive awareness and eating disorder symptoms (22), and increases the severity of suicidal ideation, whether mediated by agitation or occurring independently (23). In summary, body trust emerges as a key dimension of interoception closely linked to both eating disorders and suicidality.

Despite the significance of body trust, existing tools for measuring it are limited. In studies on body trust, the Trusting subscale of the MAIA has been widely used. However, this subscale consists of only three items and does not fully capture the multidimensional aspects of body trust. Body trust is also closely associated with positive body image and sensitivity to internal sensations (16), which can be measured using the Body Appreciation Scale-2 (BAS-2) (24) and the Visceral Sensitivity Index (VSI) (25). Nonetheless, these scales focus on only partial aspects of body trust. To overcome these limitations, Grunewald et al. (16) developed the Body Trust Scale (BTS). BTS measures body trust across three dimensions: Comfort with One’s Body (CB), Physical Attractiveness (PA), and Comfort with Internal Sensations (CIS). CB refers to trusting and feeling supported by one’s body. PA assesses the perception of one’s body as appealing in both personal and social contexts. CIS represents the ability to comfortably accept internal bodily sensations. To evaluate psychometric properties of BTS, Grunewald et al. (16) examined its factor structure, internal consistency, test-retest reliability, convergent/divergent validity, predictive validity, and measurement invariance across gender using a sample of 479 U.S. adults. The results demonstrated that the BTS has a three-factor structure, strong internal consistency, and solid validity. Test-retest reliability of BTS was significant, and measurement invariance across gender was supported.

From a cultural perspective, individuals in non-Western cultures are reported to have lower interoceptive accuracy compared to those in Western cultures (26), which may be attributed to their collectivistic thinking and context-oriented information processing styles (26, 27). A study by Lee et al. (28) examining individuals with non-psychotic depression and anxiety disorders in South Korea found that, among the MAIA subscales, the Trusting subscale showed the strongest association with anxiety. Notably, suicide is a significant social issue across all age groups in Korea (29). These findings highlight the importance of exploring the relationship between body trust and mental health within the Korean cultural context. Nevertheless, research specifically focusing on body trust in Korea remains scarce.

In addition, interoceptive ability (e.g., accuracy, sensibility) has been shown to decline with age (30, 31). For instance, Dobrushina et al. (30) reported a negative correlation between age and the Trusting subscale of the MAIA-2. This suggests the need to investigate whether the BTS functions differently across age groups. Although previous research confirmed measurement invariance of the BTS across genders (16), its measurement invariance across age groups has yet to be investigated.

Thus, our study aimed to validate a Korean version of the BTS with a Korean adult sample. We administered the BTS and other measures to a sample of Korean adults and analyzed its factor structure, reliability, and convergent/discriminant validity. Measurement invariance across age groups and gender was also examined.

## 2 Methods

### 2.1 Participants/procedure

A sample of 800 Korean adults was recruited through an online research participant system. Balanced distribution was applied based on gender and age groups: young adults (20–34 years), middle-aged adults (35–49 years), older adults (50–64 years), and seniors (65 years and over). After excluding 25 multivariate outliers identified using Mahalanobis distance (*p* <.001) (32), the final sample comprised 775 participants, which exceeds the recommended sample size (32, 33). The sample consisted of 387 women (49.9%) and 388 men (50.1%), and was distributed across age groups as follows: 195 young adults (25.2%), 197 middle-aged adults (25.4%), 191 older adults (24.6%), and 192 seniors (24.8%). The mean age of the sample was 49.34 years (*SD* = 15.26; range: 20–80 years). Participants provided informed consent and completed the survey through an online research participation system.

### 2.2 Measures

#### 2.2.1 Body Trust Scale

The Body Trust Scale (BTS) is an 11-item scale that assesses body trust across multiple dimensions (16). Each item is rated on a 7-point Likert scale, ranging from 1 (*strongly disagree*) to 7 (*strongly agree*). The original version of the BTS was translated into Korean and subsequently back-translated by the first author. Finally, the translation accuracy was verified by the authors. As part of the cultural adaptation process, the authors considered cultural characteristics of individuals in East Asian contexts, who may have difficulty attending to internal states due to their context-oriented attentional tendencies (26). To aid comprehension of physical sensations, examples such as heart rate, warmth, and satiety were included in the instructions and relevant items. These examples were based on those proposed by Grunewald et al. (3) to illustrate interoception (See Appendix).

#### 2.2.2 Multidimensional Assessment of Interoceptive Awareness

The Multidimensional Assessment of Interoceptive Awareness (MAIA) is a 32-item measure assessing interoceptive sensibility (15). Mehling et al. (15) proposed that the MAIA has an eight-factor structure. However, Gim et al. (17) found that a six-factor structure was more suitable for the Korean version (K-MAIA) and validated its psychometric properties. The six subscales include Noticing, Accept, Attention regulation, Mind-body connection awareness, Return to body, and Trusting. Noticing captures the awareness of bodily sensations. Accept measures the tendency to accept rather than suppress or avoid negative emotions in response to unpleasant bodily sensations. Attention regulation reflects the ability to manage attention toward bodily sensations, and Mind-body connection awareness assesses the recognition of the relationship between bodily sensations and emotions. Return to body is associated with directing attention to bodily sensations to achieve functional outcomes. Finally, Trusting measures the degree to which one perceives their body as safe and reliable. The items are scored on a 7-point Likert scale (from 0 to 6), and each subscale score was calculated as the mean of its items. The internal consistency for the six subscales in this study ranged from α = .81-.92.

#### 2.2.3 Body Appreciation Scale-2

The Body Appreciation Scale-2 (BAS-2) is a 10-item scale that assesses positive body image (24). Responses are scored using a 5-point Likert scale, ranging from 1 to 5. We used the Korean version of BAS-2, which has demonstrated good reliability and validity (34). In this study, the BAS-2 demonstrated high internal consistency (Cronbach’s α = .93).

#### 2.2.4 Body Investment Scale

The Body Investment Scale (BIS) consists of 24 items that assess the level of emotional, cognitive, and behavioral engagement with one’s own body (35). Participants respond on a 5-point Likert scale ranging from 1 to 5. Mok (36) translated the BIS into Korean and validated its psychometric properties. In the current sample, the internal consistency for the BIS was good (Cronbach’s α = .84).

#### 2.2.5 Visceral Sensitivity Index

The Visceral Sensitivity Index (VSI) is a 15-item self-reported measure that assesses gastrointestinal symptom-specific anxiety (25). Jang (37) translated the VSI into Korean. Items on the VSI are scored on a 6-point Likert scale, ranging from 1 (*strongly agree*) to 6 (*strongly disagree*), with total scores computed using reverse scoring. In this study, the VSI demonstrated high internal consistency (Cronbach’s α = .93).

#### 2.2.6 Eating Disorder Inventory-Very Short

The EDI-VS is a brief 16-item version of the Eating Disorder Inventory (EDI) that assesses behaviors and personality traits associated with eating disorders (38). While the EDI-VS employs a Visual Analog Scale (VAS), we adapted it to a 6-point Likert scale (ranging from 1 = *never* to 6 = *always*) following the EDI response format (39) to ensure consistency across the measurement tools used. The adapted version was then translated into Korean. In this study, the Perfectionism (PF) subscale was excluded from the total EDI-VS score. The adjusted total score and the PF subscale were then analyzed separately to explore their distinct relationships with the BTS. The internal consistency of the EDI-VS in this study was α = .70, while the PF subscale showed α = .52. However, Maiano et al. (38) noted that each subscale consisted of only two items and was limited by its small number of items. To address this issue, they applied the Spearman-Brown prophecy formula. In line with their approach, this study also applied this formula and found that the estimated reliability for the PF subscale increased to 0.76.

#### 2.2.7 Self-Harm Inventory

The Self-Harm Inventory (SHI) is a self-reported measure assessing various self-harm behaviors (40). We used a Korean version of the SHI, which was initially translated and validated by Kong (41) and subsequently modified by Lee (42) for use in the general population. The original SHI consists of 20 items, but item 13 was excluded as it overlapped conceptually with item 1 (43). Consequently, 19 items were used in this study. Responses are rated on a 4-point Likert scale, ranging from 1 (*rarely*) to 4 (*always*), and the internal consistency of the SHI in this study was high (Cronbach’s α = .93).

#### 2.2.8 Depressive Symptom Inventory-Suicidality Subscale

The Depressive Symptom Inventory-Suicidality Subscale (DSI-SS) is a subscale of the Hopelessness Depression Symptom Questionnaire, consisting of 4 items (44). This scale assesses the frequency of suicidal ideation, suicide planning, perceived controllability of suicidal thoughts, and suicide-related impulses during the past two weeks. Psychometric properties of the Korean version of the DSI-SS were validated by Suh et al. (45). The DSI-SS is rated on a 4-point Likert scale (ranging from 0 to 3). For example, a score of 0 on item 4 corresponds to “*I do not feel suicidal impulses (0 days)*”, whereas a score of 3 corresponds to “*I always feel suicidal impulses (10–14 days)*”. The internal consistency for the DSI-SS in the current sample was high (Cronbach’s α = .93).

### 2.3 Statistical analyses

To examine the factor structure of the BTS, we conducted confirmatory factor analysis (CFA) using AMOS 22.0. Goodness of fit was tested with the root mean square error of approximation (RMSEA) and the standardized root mean square residual (SRMR) (46). According to the fit criteria proposed by Schermelleh-Engel et al. (47), an RMSEA ≤ 0.05 and an SRMR ≤ 0.05 indicate a good fit, whereas an RMSEA between 0.05 and 0.08 and an SRMR between 0.05 and 0.10 indicate an acceptable fit. The comparative fit index (CFI) (48) and the Tucker-Lewis index (TLI) (49) were also reported. According to Hu and Bentler (46), CFI and TLI values greater than 0.95 indicate a good fit. We additionally reported the goodness-of-fit index (GFI) and the adjusted goodness-of-fit index (AGFI) (50) as indicators of model fit. GFI values greater than 0.95 and AGFI values greater than 0.90 were interpreted as indicative of good fit (47). Although the chi-square test of model fit was reported, it was not considered a primary fit index due to its sensitivity to sample size (47). Further, average variance extracted (AVE) was calculated to examine convergent validity and discriminant validity within the factor structure. An AVE value of .50 or higher indicates adequate convergence, and discriminant validity is supported when the square root of the AVE for each factor exceeds its inter-factor correlations (51).

To examine whether the BTS factors were consistent across age groups and gender, we conducted a measurement invariance test using multiple-group confirmatory factor analysis. Measurement invariance was examined through the following sequential process: equivalence of factor structure, factor loadings, indicator intercepts, and latent means. At each step, the changes in CFI (ΔCFI) and RMSEA (ΔRMSEA) were examined. We followed the criteria proposed by Chen (52), who suggested that when total sample size is sufficiently large (N > 300) and the group sizes are approximately equal, measurement invariance can be inferred if ΔCFI ≤ 0.01 and ΔRMSEA ≤ 0.015. The chi-square statistics were reported but not used as a primary criterion due to its hypersensitivity and tendency to over-reject models (52).

Finally, the internal consistency of the BTS was assessed using Cronbach’s alpha, McDonald’s omega, and composite reliability (CR). Two-tailed Pearson correlations were used to analyze the relations between the BTS and the other measures in the current study. These analyses were conducted using SPSS 25.0 and jamovi 2.3.28.

## 3 Results

### 3.1 Reliability of the BTS

Basic psychometric properties of the BTS items are presented in **Table 1**. All items showed adequate part-whole corrected item-total correlations and inter-item correlations. The internal consistency of the total items of the BTS was high (Cronbach’s α = .91; McDonald’s ω = .91). The CB subscale, PA subscale, and CIS subscale of the BTS also showed overall good internal consistency (Cronbach’s α = .86 -.94; McDonald’s ω = .86 -.94; CR = .86 -.94).

**Table 1:** Psychometric properties of the BTS Items (N = 775).

			IIC										
Item	Mean (*SD*)	CITC	1	2	3	4	5	6	7	8	9	10	11
1	4.77 (1.12)	.69**	–										
2	4.97 (1.09)	.65**	.76**	–									
3	4.63 (1.21)	.62**	.72**	.71**	–								
4	4.85 (1.12)	.69**	.78**	.75**	.77**	–							
5	5.02 (1.11)	.65**	.73**	.74**	.69**	.79**	–						
6	4.05 (1.41)	.61**	.33**	.31**	.24**	.30**	.29**	–					
7	4.13 (1.53)	.73**	.45**	.42**	.36**	.44**	.41**	.77**	–				
8	4.35 (1.56)	.71**	.43**	.40**	.34**	.39**	.38**	.68**	.78**	–			
9	4.41 (1.46)	.67**	.36**	.33**	.33**	.36**	.34**	.50**	.56**	.62**	–		
10	4.16 (1.43)	.60**	.31**	.24**	.29**	.31**	.30**	.45**	.48**	.50**	.68**	–	
11	3.96 (1.35)	.60**	.33**	.28**	.35**	.35**	.34**	.38**	.47**	.48**	.63**	.70**	–

BTS, Body Trust Scale; CITC, part-whole Corrected Item-Total-Correlation; IIC, Item-Inter-Correlation. **p < .01.

### 3.2 Factor structure of the BTS

**Figure 1:**
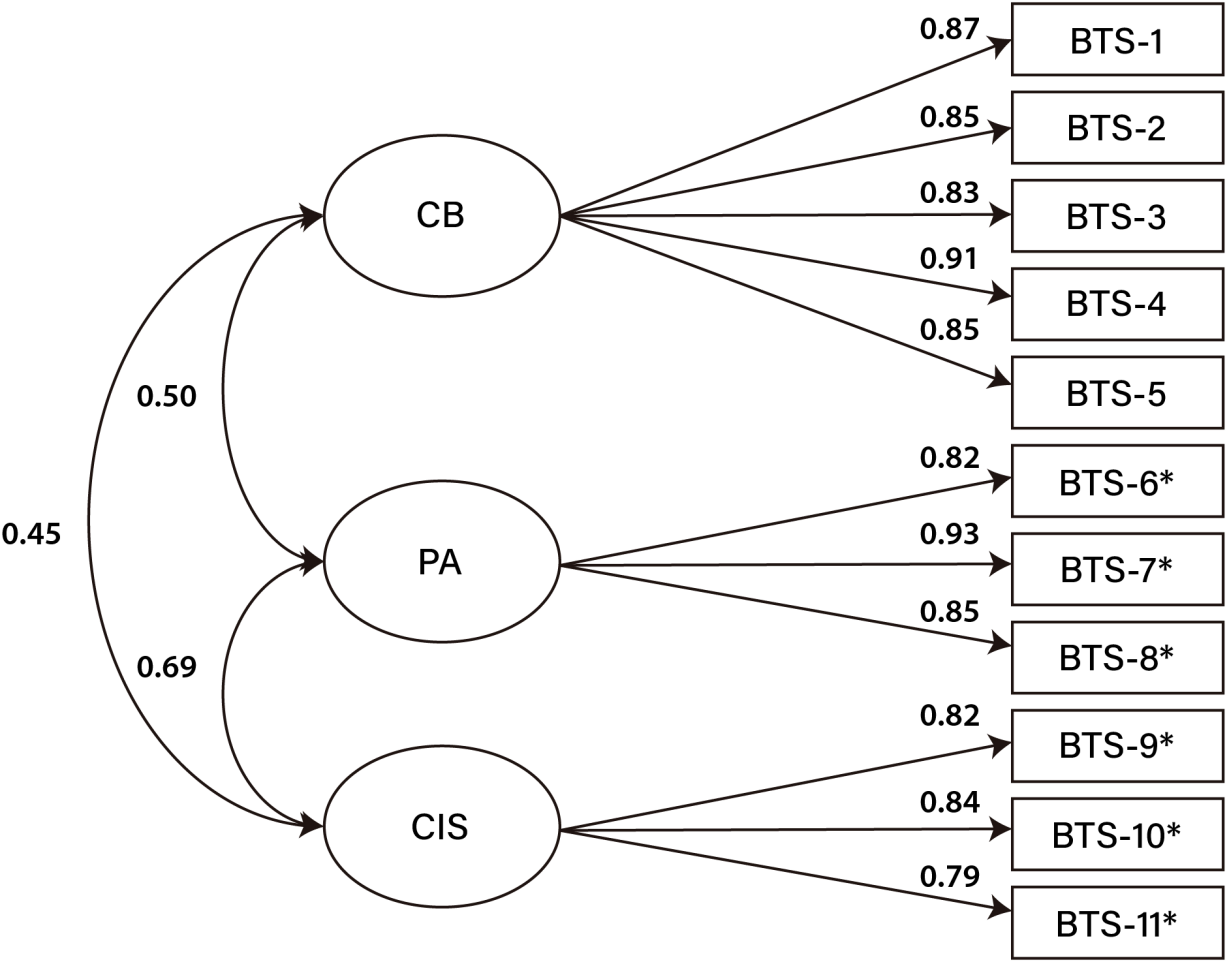
3-factor model of the BTS (N = 775). Straight arrows, standardized regression weights, curved arrows, correlations between factors. CB, Comfort with One’s Body; PA, Physical Attractiveness; CIS, Comfort with Internal Sensations. *Reverse coded items.

We conducted CFA to evaluate the original 3-factor model of the BTS proposed by Grunewald et al. (16). The model fit of the 3-factor model (*N* = 775) was acceptable [*x* ^2^ (41) = 193.452, *p* <.001; CFI = 0.976; TLI = 0.968; RMSEA = 0.069; SRMR = 0.037; GFI = 0.956; AGFI = 0.929]. The measurement model of the BTS is presented in **Figure 1**. In addition, AVE values were calculated to assess convergent validity within the factor structure. All subscales demonstrated adequate convergent validity (CB = .744; PA = .753; CIS = .667). Discriminant validity was also supported, as the square root of the AVE for each factor (CB = .863; PA = .868; CIS = .817) was greater than inter-factor correlations, from .45 to .69 (**Figure 1**).

Based on the relatively high part-whole corrected item-total correlations (**Table 1**) and inter-factor correlations (**Figure 1**), we additionally explored two alternative models: a unidimensional model and second-order factor model. The unidimensional model showed substantially poorer fit, whereas the second-order factor model demonstrated an identical level of model fit to the 3-factor model (see **Supplementary Material Table 1**). However, since the original scale (16) adopted a 3-factor model, the second-order factor model did not show a better fit than the 3-factor model, and each subscale is theoretically and practically meaningful, we retained the original 3-factor model as the primary structure for this study.

### 3.3 Invariance Testing

#### 3.3.1 Measurement invariance across age groups

First, the configural invariance model was tested across age groups. Specifying the same factor structure simultaneously across age groups demonstrated an overall good fit, supporting factor structure equivalence [*x* ^2^ (164) = 328.056, *p* <.001; CFI = 0.975; TLI = 0.966; RMSEA = 0.036; SRMR = 0.032; AIC = 616.056]. Second, the equivalence of factor loadings was tested across age groups. The analysis confirmed equivalent factor loadings, as evidenced by ΔCFI = - 0.001 and ΔRMSEA = - 0.002 [*x* ^2^ (188) = 355.676, *p* <.001; CFI = 0.974; TLI = 0.970; RMSEA = 0.034; SRMR = 0.033; AIC = 595.676]. Third, the equivalence of the intercepts was tested across age groups. Intercept equivalence was not supported (ΔCFI = -0.014; ΔRMSEA = 0.005), as ΔCFI exceeded the cutoff. This suggests that one or more parameters differed across age groups. When the intercepts of item 9 were freely estimated, partial scalar invariance could be demonstrated [*x* ^2^ (218) = 448.188, *p* <.001; CFI = 0.965; TLI = 0.964; RMSEA = 0.037; SRMR=0.035, AIC = 628.188]. The estimates of intercepts of item 9 across age groups were 4.15 (young adults), 4.27 (middle-aged adults), 4.46 (older adults) and 4.65 (seniors). This suggests that item 9 may be interpreted differently across age groups. Fourth, the equivalence of the latent means was tested across age groups. The change in CFI and RMSEA (ΔCFI = 0.004; ΔRMSEA = - 0.002) supported latent mean equivalence.

#### 3.3.2 Measurement invariance across gender

Gender invariance was tested following the same four-step procedure used for age groups (i.e., configural, metric, scalar, and latent mean invariance). All levels demonstrated acceptable fit indices and changes within the predefined thresholds (ΔCFI ≤ 0.01; ΔRMSEA ≤ 0.015), supporting full measurement invariance across gender. Detailed fit statistics are provided in the **Supplementary Material Table 2**.

### 3.4 Correlations between the BTS and other measures

All subscales of the BTS were closely related to the Trusting subscale of the K-MAIA. The CB factor was strongly correlated with most K-MAIA subscales (e.g., Noticing, Attention regulation, Mind-body connection awareness, Return to body, and Trusting), with the correlation being highest for the Trusting subscale. Positive body image and body investment also demonstrated strong associations, particularly with the CB factor. The PA factor was closely related to body investment and showed the strongest negative correlation with eating pathology among the BTS subscales. The CIS factor exhibited the highest negative correlation with visceral sensitivity. Weak but significantly negative correlations were observed between all BTS factors and self-harm behaviors as well as suicidal ideation. Perfectionism was either not significant or weakly related to all BTS factors (see **Table 2**).

**Table 2:** Correlation between the BTS and other measures (N = 775).

Measures	BTS-CB	BTS-PA	BTS-CIS
Noticing	.47**	.24**	.13**
Accept	.15**	-.12**	.01
Attention regulation	.57**	.26**	.27**
Mind-body connection awareness	.53**	.28**	.19**
Return to body	.57**	.25**	.23**
Trusting	.74**	.38**	.34**
BAS-2	.74**	.48**	.38**
BIS	.62**	.61**	.49**
VSI	-.24**	-.34**	-.41**
EDI-VS	-.43**	-.50**	-.42**
EDI-VS PF	.25**	.02	-.01
SHI	-.11**	-.21**	-.20**
DSI-SS	-.25**	-.24**	-.20**

BTS-CB, Body Trust Scale-Comfort with One’s Body; BTS-PA, Body Trust Scale-Physical Attractiveness; BTS-CIS, Body Trust Scale-Comfort with Internal Sensations; Noticing, Noticing subscale of the K-MAIA; Accept, Accept subscale of the K-MAIA; Attention regulation, Attention regulation subscale of the K-MAIA; Mind-body connection awareness, Mind-body connection awareness subscale of the K-MAIA; Return to body, Return to body subscale of the K-MAIA; Trusting, Trusting subscale of the K-MAIA; BAS-2, Body Appreciation Scale-2; BIS, Body Investment Scale; VSI, Visceral Sensitivity Index; EDI-VS, Eating Disorder Inventory-Very Short; EDI-VS-PF, Eating Disorder Inventory-Very Short-Perfectionism; SHI, Self-Harm Inventory; DSI-SS, Depressive Symptom Inventory-Suicidality Subscale; K-MAIA, Korean version of Multidimensional Assessment of Interoceptive Awareness. ** *p* <.01.

## 4 Discussion

The aim of this study was to validate the Korean version of the BTS and evaluate its psychometric properties in a Korean sample. The results showed that the items of the BTS exhibited strong internal consistency. As Grunewald et al. (16) suggested, the 3-factor model achieved satisfactory fit. Convergent and discriminant validity within the factor structure were also supported. This indicates that the three dimensions of body trust are present and function similarly within Korean culture. Although the 3-factor model was adopted as the main model in this study, the presence of a second-order factor—labeled ‘body trust’—was also confirmed. This finding suggests that, in addition to subscale scores, the total score of the BTS can also be meaningfully used.

To examine whether the factor structure of the BTS differs across age groups and gender, measurement invariance was tested in the current sample. First, age groups were examined. Full metric invariance was established, indicating that the factor loadings are equivalent across age groups. However, full scalar invariance was not achieved. Instead, partial scalar invariance was confirmed with the free estimation of item 9 (“Physical sensations in my body make me nervous/uncomfortable.”). Given that item 9 is reverse-coded, its intercepts decreased across age groups in the following order: young adults, middle-aged adults, older adults, and seniors. Since item 9 assesses emotional responses to bodily sensations, this finding may be consistent with a previous study (53) reporting a weaker connection between interoception and emotional reactivity in older individuals compared to younger individuals.

There are several possible explanations for this result. Older individuals tend to accept bodily sensation abnormalities as a natural part of the aging process, whereas younger individuals regard it as unusual and therefore experience more anxiety (54). Another possible explanation is that older individuals focus on emotional well-being and tend to minimize negative emotions, influenced by the recognition that their remaining time in life is finite (55). This interpretation is also supported by findings from a Korean study conducted with older adults (56). These results suggest the potential need for revising item 9. Future research may consider rewording this item or developing age-appropriate versions of the item.

Based on the partial scalar invariance model, the analysis of latent mean equivalence revealed that there were no significant mean differences in the BTS scores across age groups. This finding contrasts with a previous study (30) and indicates that in Korean culture, body trust levels do not significantly vary across age groups. Two hypotheses may explain this result. First, the discrepancy may stem from differences in measurement tools. The previous study assessed body trust using the Trusting subscale of the MAIA, which, as mentioned earlier, focuses on a narrow aspect of body trust. In contrast, this study employed the BTS, which includes CB, PA, and CIS to measure body trust. Therefore, to better understand these differences in findings, it is necessary to consider and control for the impact of measurement tool differences. Future research should re-examine age-related body trust levels in Western cultures using the BTS. Second, interoceptive accuracy (the ability to objectively detect internal sensations) and interoceptive sensibility (subjective belief in one’s ability to perceive and process internal sensations) are dissociated dimensions (7, 57). The relationship between these two dimensions may vary across cultures. In particular, these two dimensions may be negatively associated in non-Western cultures (57) due to collectivistic thinking and a tendency toward context-oriented information processing (26, 27). In other words, the tendency to prioritize communal and social harmony over individual internal states may influence this relationship (58). Although no studies have specifically addressed this issue in Korean culture, previous research allows for the following inference. As in Western cultures (30, 31), interoceptive accuracy may have declined with age in Korean culture. However, if the negative relationship between these two dimensions preserved interoceptive sensibility despite the decline in interoceptive accuracy, body trust levels may have remained stable. In future research, it would be meaningful to longitudinally track how body trust levels change throughout an individual’s developmental process while considering cultural influences. Another valuable avenue for research would be to explore how the relationships among different dimensions of interoception evolve over time in Korean culture.

Following this, measurement invariance across gender was tested. The results replicated the findings of Grunewald et al. (16), indicating that the factor structure of BTS functions equivalently for men and women in the Korean context as well.

Correlation analysis confirmed the convergent validity of the Korean version of the BTS. First, the results showed that the CB factor was strongly associated with most K-MAIA subscales: Noticing, Attention regulation, Mind-body connection awareness, Return to body, and Trusting. Positive body image and body investment were also closely related. The CB factor, in particular, showed a stronger association with the K-MAIA Trusting subscale compared to the other BTS factors. These findings are consistent with a previous study (16) indicating that the CB factor is particularly related to existing measures of body trust. Second, the PA factor was related to body investment and positive body image. Our expectations were based on previous findings by Grunewald et al. (16), which reported negative associations between body dissatisfaction and the PA factor, and by Mok (59), which demonstrated positive associations between appearance appreciation and body investment. These results are consistent with those expectations. Third, the CIS factor showed the most notable negative correlation with visceral sensitivity among the factors of BTS. This finding also aligns with a previous study (16), reaffirming that the CIS factor measures anxiety and hypersensitivity related to internal bodily sensations. Fourth, all three factors of the BTS showed weak but significant correlations with self-harm behaviors and suicidal ideation. This is consistent with previous studies (10, 16) that have suggested that body trust is related to self-harm and suicidal ideation. Regarding its relationship with eating pathology, all three BTS factors showed significant negative correlations with the EDI-VS scale. This is also consistent with previous studies (20, 21) that have reported a negative relationship between body trust and eating disorders. Among the BTS factors, the PA factor was the most closely associated with EDI-VS. These results indicate that higher perceived physical attractiveness is linked to lower levels of eating pathology. In summary, these findings support the convergent validity of the BTS with related variables.

For the discriminant validity of the BTS, a weak but significant correlation was found between Perfectionism and the CB factor. However, no significant correlations were observed with the other BTS factors. This result is aligned with previous studies (39) that found no significant relationship between interoceptive awareness and perfectionism, both of which are subscales of the EDI. Therefore, this finding supports the discriminant validity of the BTS. Taken together, the findings discussed above confirm that the BTS is a reliable and valid tool for measuring body trust.

### 4.1 Limitations

This study has several limitations to consider. First, although body trust has been identified as a relatively stable construct (16), this study was cross-sectional and did not investigate the temporal stability of the BTS. Future research should examine the test–retest reliability of the scale. Second, although body trust is associated with eating pathology, self-harm behaviors, and suicidal ideation, this study was conducted with a non-clinical population. Additionally, since this study was conducted with adults, its findings may not be applicable to adolescents. However, failure to adequately interpret and process interoceptive signals during adolescence, a period of significant physical and psychological changes, may increase the risk of developing psychopathology (5). Thus, it would be valuable to validate the BTS with clinical populations and adolescents. Third, because this study used an online research participant system, the sample, especially seniors, may be biased toward those more familiar with using the internet. Future research should include individuals with lower digital accessibility to ensure a more diverse and representative sample. Lastly, this study established the convergent validity of the BTS with related constructs. However, alexithymia is associated with atypical interoception (60, 61) and could serve as a useful construct for verifying the convergent validity of the BTS. Therefore, it would be meaningful to re-examine the convergent validity of the BTS by including alexithymia as well as other potentially related constructs.

## 5 Conclusion

Our study is the first to examine the reliability and validity of a Korean version of the BTS, a recently developed tool designed to comprehensively measure body trust. The large sample size and the balanced distribution of age and gender groups strengthen the validity of our findings. In conclusion, while further validation in clinical and adolescent population is warranted, the Korean version of the BTS appears to be a sound instrument with potential applicability in Korean adults. Furthermore, it may serve as a valuable tool for exploring associations between body trust and mental health factors, such as eating pathology and suicide risk (16).

## Data Availability

The raw data supporting the conclusions of this article will be made available by the authors, without undue reservation.

## Appendix. The Korean version of the BTS

Instructions; 다음은 귀하가 신체와 그 감각에 대해 가질 수 있는 다양한 태도, 감정 또는 행 동을 알아보기 위한 문항입니다. 아래 각 문항에 대해 얼마나 동의하시는지 해당하는 숫자에 표시해주십시오.

*9~11번 문항의 ‘신체 감각’ 이란, 심장박동, 포만감, 열감, 내장 감각 등 신체 내부에서 느낄 수 있는 감각을 의미합니다.

**Table:**

1	2	3	4	5	6	7
전혀 그렇지 않다			보통이다	매우 그렇다		
1. 내 몸은 믿을만하다.						
2. 내 몸은 나에게 도움을 준다.						
3. 내 몸은 내가 원하는 대로 기 능한다.						
4. 내 몸을 신뢰할 수 있다.						
5. 나는 일상생활에서 내 몸이 제 역할을 해낼 거라고 믿는다.						
6. 내 몸은 사회가 매력적이라고 여기 는 기 준에 맞지 않는다.						
7. 나는 개인적으로 내 몸이 매력적이라고 생각하지 않는다.						
8. 나는 내 몸의 크기 와 외형이 불편하다.						
9. 나의 신체 감각(심장박동, 포만감, 열감 등) 때문에 불안하거나 불편해진다.						
10. 나의 신체 감각(심장박동, 포만감, 열감 등)은 나에게 어떤 문제가 있다는 생각이 들게 한다.						
11. 내 몸에서 신체 감각(심장박동, 포만감, 열감 등)이 느껴질 때, 그것을 즐기 기 어렵다.						
