# Supplementary material for: Body trust in Korean population: validation of the Korean version of the body trust scale
Source: Front Psychiatry. 2025 Aug 21;16:1631918. doi: 10.3389/fpsyt.2025.1631918 (PMC12410140; doi:10.3389/fpsyt.2025.1631918)
Supplement: Supplementary file 1 [file Table1.docx]

Supplementary Material

# Supplementary Table 1. Model fit indices for alternative models of the BTS

|  | $x^{2}$(*df*) | CFI | TLI | RMSEA | SRMR | GFI | AGFI |
| --- | --- | --- | --- | --- | --- | --- | --- |
| Unidimensional model | 2347.492 (44), *p*<.001 | 0.642 | 0.552 | 0.260 | 0.180 | 0.559 | 0.339 |
| Second-order factor model | 193.452 (41), *p*<.001 | 0.976 | 0.968 | 0.069 | 0.037 | 0.956 | 0.929 |

*Note*. *df*: degree of freedom; CFI: Comparative Fit Index; TLI: Tucker-Lewis Index; RMSEA: Root Mean Square Error of Approximation; SRMR: Standardized Root Mean square Residual; GFI: Goodness-of-Fit Index; AGFI: Adjusted Goodness-of-Fit Index.

# Supplementary Table 2. Measurement invariance across gender

|  | $x^{2}$(*df*) | CFI | TLI | RMSEA | SRMR | ΔCFI | ΔRMSEA |
| --- | --- | --- | --- | --- | --- | --- | --- |
| Configural invariance | 228.111 (82), *p*<.001 | 0.977 | 0.969 | 0.048 | 0.030 | N/A | N/A |
| Metric invariance | 243.350 (90), *p*<.001 | 0.976 | 0.971 | 0.047 | 0.031 | -0.001 | -0.001 |
| Scalar invariance | 266.030 (101), *p*<.001 | 0.974 | 0.972 | 0.046 | 0.031 | -0.002 | -0.001 |
| Latent mean invariance | 261.224 (98), *p*<.001 | 0.975 | 0.971 | 0.046 | 0.031 | -0.001 | 0.000 |

*Note*. *df*: degree of freedom; CFI: Comparative Fit Index; TLI: Tucker-Lewis Index; RMSEA: Root Mean Square Error of Approximation; SRMR: Standard Root Mean Square; ΔCFI and ΔRMSEA refer to changes compared to the previous model.
